# Supplementary figures and images for: Thermopneumatic suction integrated microfluidic blood analysis system
Source: PLoS One. 2019 Mar 7;14(3):e0208676. doi: 10.1371/journal.pone.0208676 (PMC6405101; doi:10.1371/journal.pone.0208676)

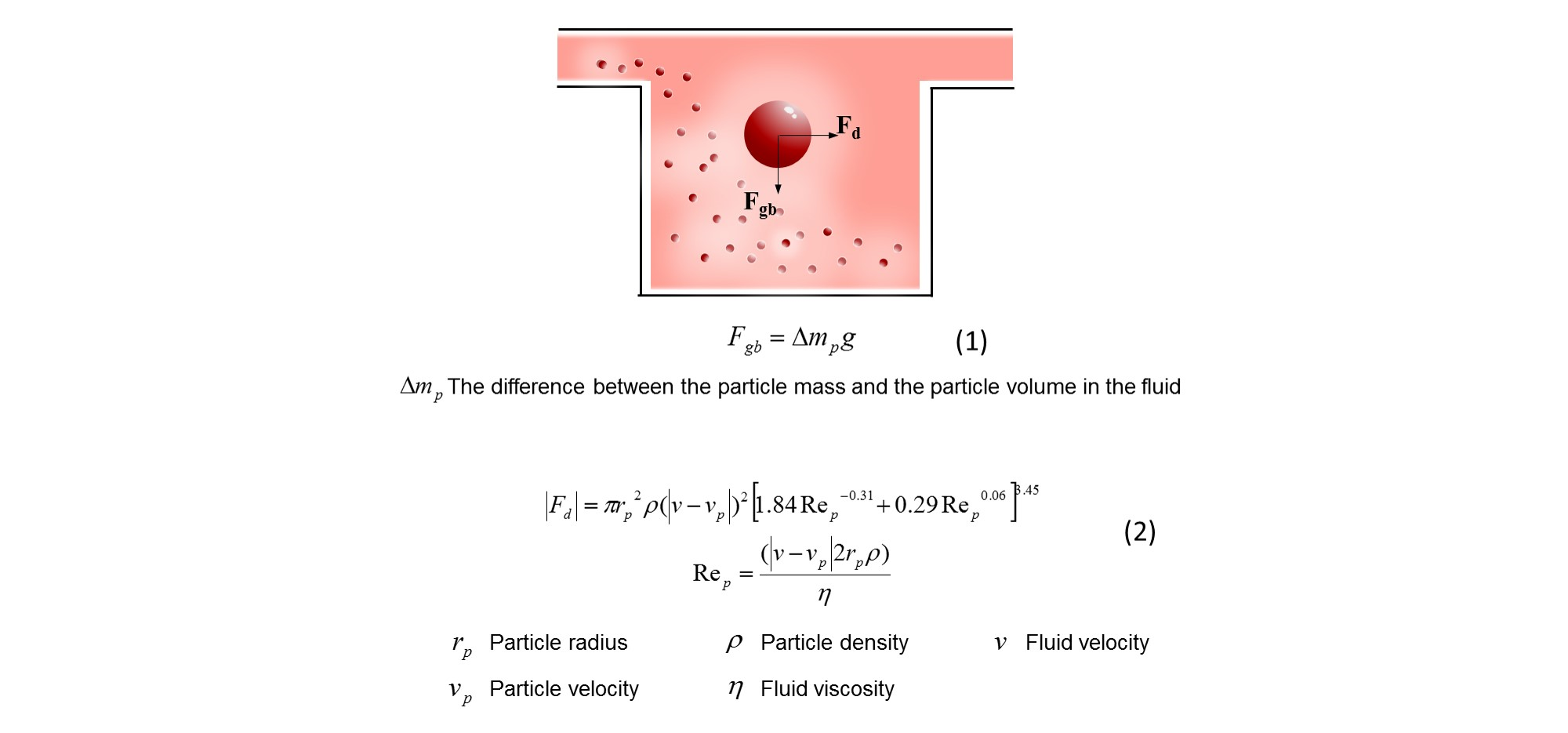

Supplement: S1 Fig — Modeling the principle force acting on a suspended particle, for example, the blood cell in the filter trench. The relevant forces are buoyancy-corrected gravitational sedimentation force Fgb and fluid drag force Fd. (TIF) [file pone.0208676.s001.tif]

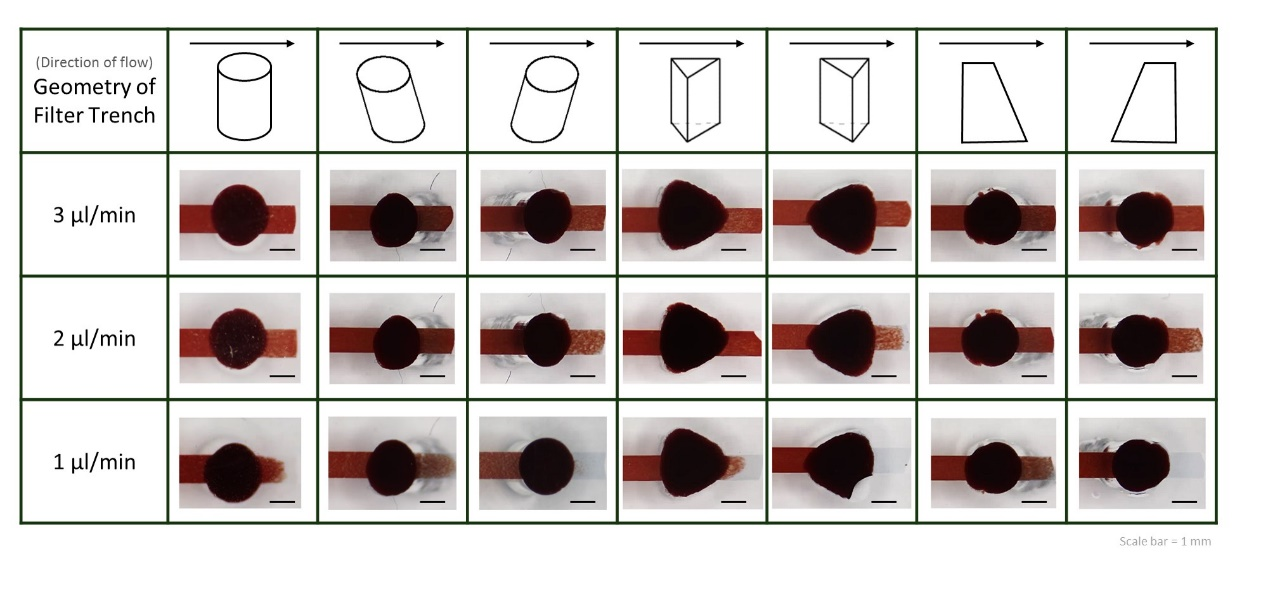

Supplement: S2 Fig — Separation efficiency in different geometries. e designed the cylindrical, tilted cylindrical, triangular prismatic, and trapezoidal columnar trenches to examine the separation efficiency in different geometries. The filtered plasma was expected to be transparent. The appearance of a reddish‐brown fluid at the outlet of the filter trench indicated the presence of RBCs, which was defined as a failed separation. After successful plasma separation, we analyzed and calculated the separation efficiency in ImageJ. Although the triangular prismatic and trapezoidal columnar trenches showed high performance of separation, we did not use these geometry structures, because of the complexity of their fabrication. (TIF) [file pone.0208676.s002.tif]

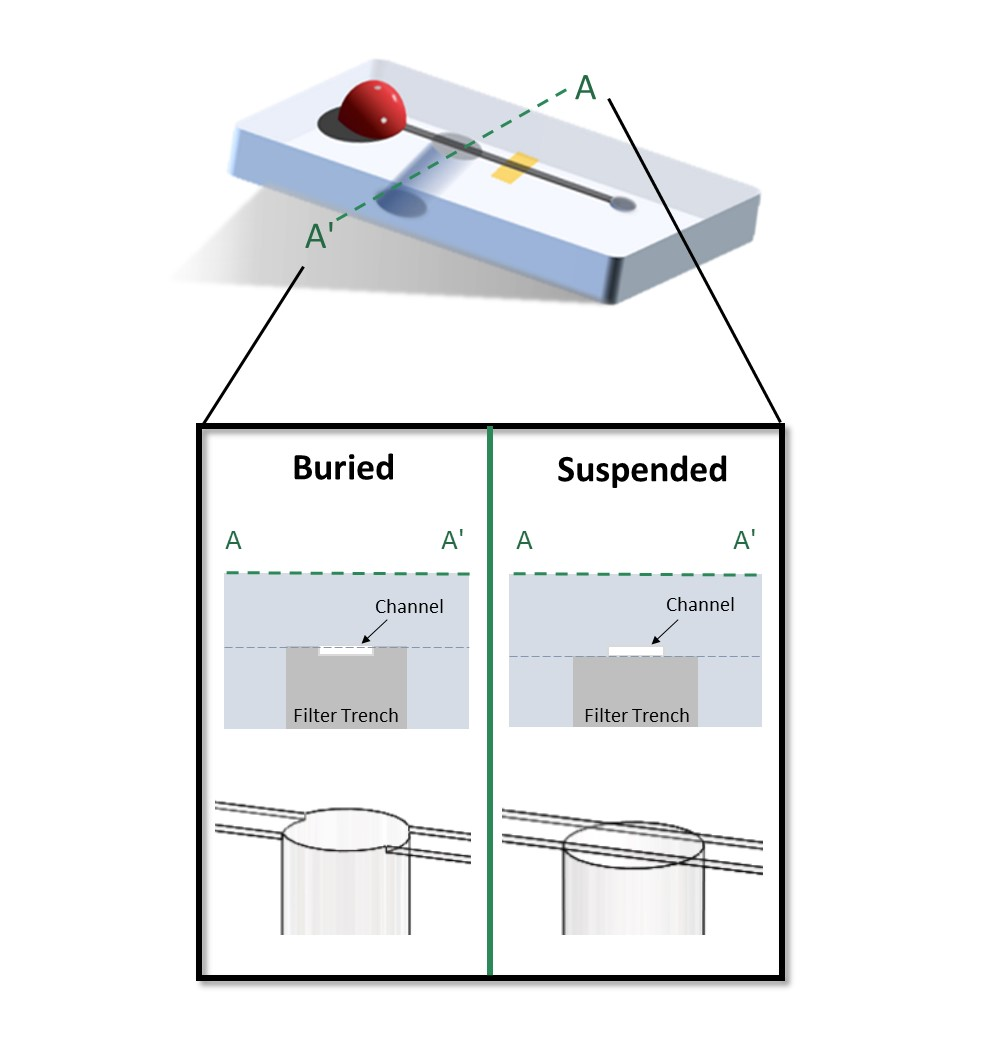

Supplement: S3 Fig — Illustration of the cross section of the buried and suspended channels. The line AA’¯ cuts across the filter trench and shows the structure of the microchannel in cross section. (TIF) [file pone.0208676.s003.tif]

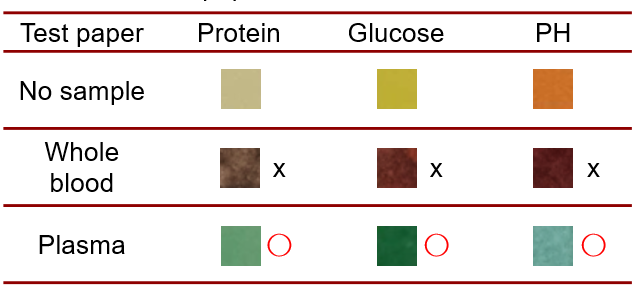

Supplement: S4 Fig — Bioassays with RBCs contained and removed sample on test paper. Chart showing the need for blood separation before examination by using biomarkers. If RBCs are present in the sample, observing the color reaction on the test paper is difficult because of the interference of hemoglobin. (TIF) [file pone.0208676.s004.tif]

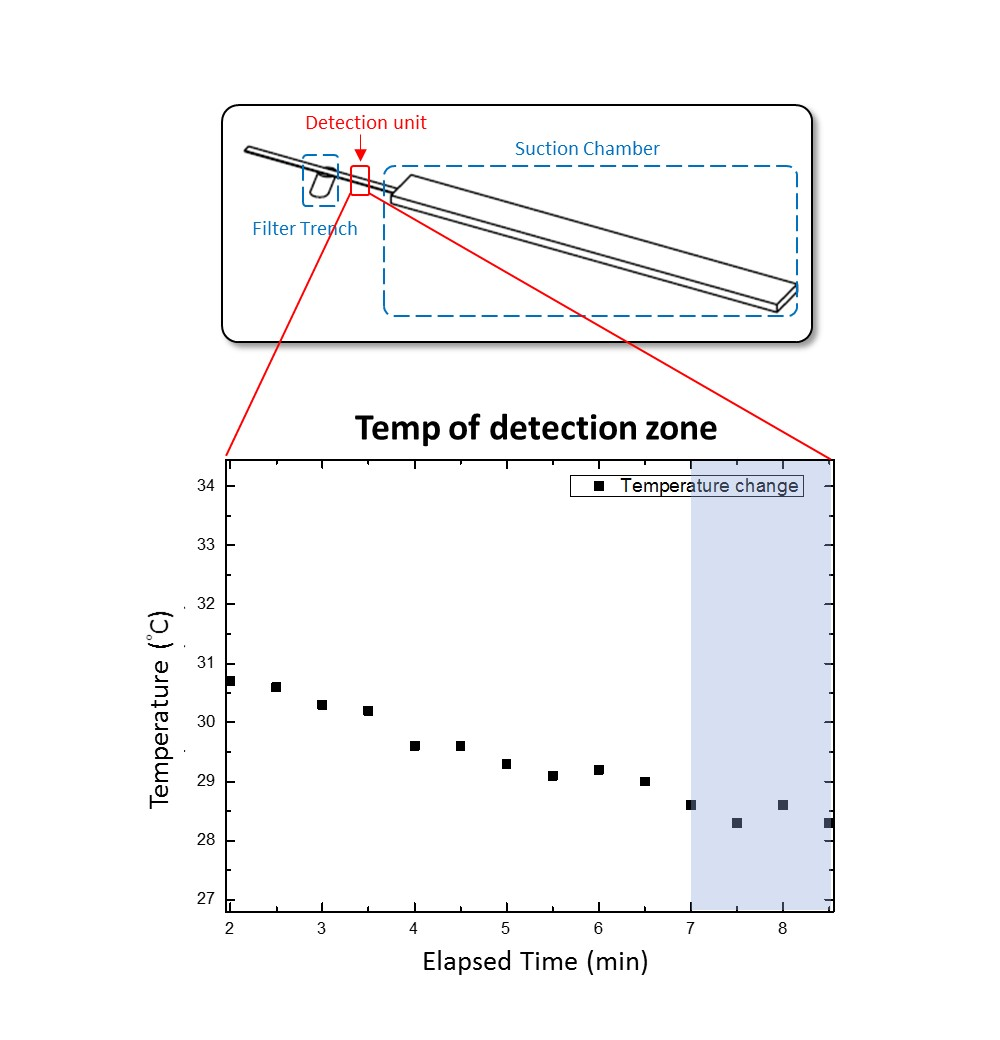

Supplement: S5 Fig — Chart showing temperature variation in the detection zone of the microfluidic chip. The temperature changed by <0.5˚C when the isolated plasma was sucked in to in the detection zone (blue area). Therefore, the sample or analytes were not denatured by the heating‐force mechanism. The elapsed time was measured from the time the heater strip was switched on to heat the suction chamber. (TIF) [file pone.0208676.s005.tif]
